# Assessing the coverage and timeliness of coronavirus vaccination among people experiencing homelessness in Wales, UK: a population-level data-linkage study

**DOI:** 10.1186/s12889-023-16432-x

**Published:** 2023-08-05

**Authors:** Ian Thomas, Peter Mackie

**Affiliations:** 1 Administrative Data Research Wales/Cardiff University, School of Social Sciences, SPARK, Maindy Road, CF24 4HQ Cardiff, UK; 2 Cardiff University, School of Geography and Planning, Glamorgan Building, King Edward VII Avenue, CF10 3WA Cardiff, UK

**Keywords:** Homelessness, COVID-19, Vaccination, Competing risk, Survival analysis

## Abstract

**Background:**

People experiencing homelessness have elevated morbidity, increasing their risk of COVID-19 related complications and mortality. Achieving high vaccination coverage in a timely manner among homeless populations was therefore important during the mass vaccination programme in Wales to limit adverse outcomes. However, no systematic monitoring of vaccinations among people experiencing homelessness in Wales has been undertaken.

**Methods:**

Retrospective cohort analysis was conducted using de-identified administrative data. Study cohort members were adults (≥ 18 years old) living in Wales on the 2 December 2020 and who had recently experienced homelessness, defined as experiencing homelessness between 1 July 2020 and 2 December 2020. The outcome of interest was first coronavirus vaccine dose. Follow-up started on 2 December 2020, and ended if the participant died, had a break in address history > 30 days, reached the end of follow up (30 November 2021), or had the outcome of interest. Median-time-to-vaccination was used as a crude measure of ‘timeliness’ of vaccine uptake. To account for competing risk of death prior to vaccination, vaccine coverage was described using cumulative incidence at 350-days, and at 50-day increments over follow-up (2 December 2020 to 17 November 2021). As a benchmark, all time-to-event measures were generated for the adult population in Wales with similar baseline individual and residential characteristics as the study cohort.

**Results:**

1,595 people with recent experiences of homelessness were identified and included in analysis. The study cohort were disproportionately male (68.8%) and concentrated in the most deprived areas in Wales. Median time-to-vaccination for the study cohort was 196 days (95% CI.: 184–209 days), compared to 141 days (95% CI.: 141–141 days) among the matched adult population in Wales. Cumulative incidence of vaccination after 350-days of follow-up was 60.4% (95% CI.: 57.8–62.8%) among the study cohort, compared to 81.4% (95% CI.: 81.3–81.5%) among the matched adult population. Visual analysis of cumulative incidence over time suggests that vaccine inequality, i.e., difference between study cohort and matched adult population, peaked after 200-days of follow-up, and declined slightly until last follow-up at 350-days.

**Conclusions:**

Despite being prioritised for vaccination, people experiencing homelessness in Wales appear to have been under-engaged, leading to lower vaccination coverage and greater time unvaccinated, potentially increasing their risk of COVID-19 complications and mortality.

## Background

Homelessness is a global issue, with an estimated 150 million people experiencing homelessness worldwide [1]. One of the effects of the COVID-19 pandemic was in framing homelessness as a public health concern [2–4], thereby recognising the large body of evidence highlighting worse health outcomes among homeless populations. Experiencing homelessness is associated with increased morbidity such as infections, cardiovascular and respiratory conditions [5], as well as mental health and substance use related issues [6, 7]. Mortality is also higher among people experiencing homelessness compared to housed populations [8–11]. However, homelessness is not an homogenous experience [12], and morbidity and mortality have been found to vary across different experiences of homelessness [13]. Overlapping forms of social exclusion amongst people with more ‘complex’ homelessness experiences [14, 15] can lead to greater health inequalities [16], whilst people in homeless shelters with shared airspace are at an increased risk of communicable infectious diseases such as tuberculosis, MRSA [17, 18], and COVID-19 [19].

The intersection of homelessness with complex health issues that can lead to COVID-19 related complications and mortality [20, 21], in combination with living conditions that can lead to high exposure to infectious diseases, resulted in people experiencing homelessness being considered an at-risk group in public health discourses during the pandemic [4]. Vaccinating people experiencing homelessness in a timely manner was therefore important during mass vaccination programmes across the globe. However, evidence to date has found disparities in vaccination rates between homeless and wider populations.

A study comparing vaccine uptake up to August 2021 in 6 US districts, found lower vaccine rates among people experiencing homelessness compared to the general population, ranging from 9.0 to 39.3% point difference [22]. Lower vaccination rates have also been found among Veterans experiencing homelessness in the US—45.8% compared to 64.3% for the general adult population [23]. A study based in Ontario using population level analysis of people recently experiencing homelessness, found that 61·4% of the homeless group had received their first dose of the COVID-19 vaccine compared to 86·6% of adults in Ontario [24]. To date, no studies of vaccination uptake among people experiencing homelessness have been conducted in Wales.

### Public health policy response to vaccinations in Wales

The Medicines and Healthcare products Regulatory Agency in the UK granted approval for the use of the Pfizer/BioNTech vaccine on the 2 December 2020, thereby enabling mass vaccination programmes in Wales, and the UK, to start. Vaccines were initially prioritised to people most at risk, based on advice from the Joint Committee on Vaccination and Immunisation. The Joint Committee's guidance included 9 priority groups, stratified by age, presence of conditions that placed people at increased clinical vulnerability, and occupation, i.e., care and healthcare workers [25]. The Wales Immunisation System formed the basis by which people in Wales were identified and invited for vaccination, and was constructed based on lists of people registered with healthcare providers in Wales—largely primary healthcare providers. Invitation letters and mobile text messages were used to provide people with the dates and times for appointments at mass vaccination centres [26].

Guidance was released by the Welsh Government on the 10 March 2021, to the effect that people who were currently or who had recently experienced homelessness were to be prioritised for vaccination [27]. People experiencing homelessness were added to priority group 6, which included adults aged 16 to 65 years old in a clinically at-risk group. Practitioners were given discretion in how homelessness was defined. Local health boards, which oversaw the delivery of the vaccination programme, were encouraged to utilise local knowledge of the third sector and housing providers to identify people. A principal element of the guidance was an acknowledgement that people experiencing homelessness may not be able to access medical services, and that attempts should be made to take vaccines to this group of people.

Though people experiencing homelessness were prioritised for vaccination in Wales, there have been no follow up studies to determine if prioritisation occurred, i.e., vaccinations were timely, and no evidence has been produced of levels of vaccination coverage achieved among this vulnerable group.

## Methods

### Study design and data sources

A retrospective population-based cohort analysis was conducted using de-identified administrative data from healthcare and substance use services in Wales, UK. Data were accessed via the Secure Anonymised Information Linkage (SAIL) Databank. Data sets used in this analysis included: Patient Episode Database for Wales; Welsh Longitudinal General Practice dataset; Substance Misuse Data Set; the Welsh Demographic Service; and the COVID Vaccination Dataset. All data sets were linked using a unique identifier for each person in Wales, the Anonymised Linkage Field (ALF), which is assigned to all data within the SAIL Databank [28]. ALFs are assigned either deterministically, based on NHS Wales numbers, or probabilistically based on personal identifiers, such as name, date of birth, gender, and postcode. Records were retained where ALF-match accuracy was greater than 90%, or where ALFs were matched deterministically.

### Participants

The study cohort included all adults (age 18 ≥ years old) living in Wales on 2 December 2020, who had recent experiences of homelessness—defined as experiencing homelessness between 1 July 2020 and 2 December 2020. The Welsh Demographic Service was used to determine residency; being a database of addresses constructed when people register with General Practitioners in Wales. We adopted previously validated diagnosis codes and direct measures of housing need to identify people with recent experiences of homelessness from healthcare and substance use services data [29–32].

Within the Patient Episode Database for Wales and Welsh Longitudinal General Practice dataset, experiences of homelessness were identified using diagnoses codes. Use of diagnosis fields may lead to an under-representation of homelessness, as clinicians may not code homelessness unless it is diagnostically related to the healthcare incident. The Substance Misuse Data Set included responses to several questions related to housing need, which align to experiences of homelessness, i.e., living on the streets or in hostels. People accessing substance use services are asked housing need questions at initial assessment, and at 12-weekly intervals whilst engaging with services—both measures of housing need were used in this analysis. Experiences of homelessness measured in this study align with international definitions of extreme homelessness [12]—being primarily people living-on-the-streets (‘roofless’), people living in hostels, shelters, and other temporary accommodation such as Bed and Breakfasts (‘houseless’), and people in insecure housing situations, such as ‘sofa surfers’.

### Outcomes

The primary outcome was time to first recorded coronavirus vaccination, obtained from the COVID Vaccination Dataset covering all vaccinations administered (or planned) that occurred in NHS settings or were funded by the NHS in Wales. Of 7,965,716 records within the COVID Vaccination Dataset provided by SAIL, 18,071 (0.2%) were missing an ALF, and were therefore unavailable for use in linkage-based studies. For each person (ALF), date of first recorded vaccination was extracted. Follow-up started on 2 December 2020, and ended if the participant died, reached the end of follow up (30 November 2021), had a break in housing histories > 30 days, or had the outcome of interest. Breaks in residential histories were determined using the Welsh Demographic Service, which provides a history of anonymised residences, known as the Residential Anonymised Linkage Field (RALF) [33], along with people’s entry and exits from those residences.

### Statistical analysis

The study cohort were initially described in terms of individual and area characteristics, including: age (single year); gender; area deprivation measured using quartiles of the 2019 edition of the Welsh Index of Multiple Deprivation (WIMD); and Local Health Board (LHB) of residence. Means, with standard deviations, and proportions were used, where appropriate. Descriptive characteristics were chosen based on the existing literature—drawing on not-homeless populations in Wales [26, 34–36] and homeless populations outside of the UK [24]—which suggest their association with vaccine uptake. Area-based characteristics, i.e., 2019 WIMD quartile and LHB, were assigned to people based on their areas of residence at the start of follow-up (2 December 2020), obtained from the Welsh Demographic Service. For highly mobile people, which may include people experiencing homelessness, address information may not be current; This limitation has also been noted by others [24].

Median time-to-vaccination in days, being the time from study entry at which 50% of the study cohort had received their first vaccination dose, was calculated as an initial crude measure of timeliness of vaccinations. Vaccine coverage was assessed using the cumulative incidence function [37], to account for competing risk of death prior to vaccination. Cumulative incidence was calculated and plotted at 50-day increments from 2 December 2020, up to 350-days (17 November 2021). Vaccination prioritisation for people experiencing homelessness took effect from the 10 March 2021; this timepoint has been labeled in the cumulative incidence plot ('PEH prioritised for vaccination') to provide a point of reference.

To contextualise measures of median time-to-vaccination and cumulative incidence, we provide the same information for a sub-set of the adult population in Wales (n = 756,332) of similar age, gender, WIMD, and LHB as the study cohort at the start of follow-up (‘matched population’).

## Results

1,595 people were identified as being in scope for this analysis and matched the definition for having recently experienced homelessness (‘study cohort’). The characteristics of the study cohort conformed to those obtained from previous research using these, and similar, data sources [31, 32], in that they were largely male (68.2%) and concentrated in more deprived areas of Wales, with 50.2% having lived in the most deprived quartile in Wales at baseline. The average age of the study cohort was 39.4 years old (SD.: 12.8).

904 people (56.7%) in the study cohort had received at least one dose of the coronavirus vaccine by 30 November 2021. 23 people experienced the competing event of death before vaccination/loss to follow-up. Cohort members who were vaccinated did not differ to unvaccinated cohort members in terms of gender, WIMD area deprivation and LHB of residence (Table 1). However, study cohort members who were vaccinated were older compared to unvaccinated cohort members—mean age of 41.2 years old (SD.: 13.2) versus 36.7 years old (SD.: 11.5), respectively.

**Table 1:** Baseline characteristics of study cohort, split by receipt of coronavirus vaccine doses by 30 November 2021 (number and percent, unless otherwise stated)

		All study cohort members	No vaccine doses	At least one vaccine dose
Mean age, single year (SD)		39.4 (12.8)	36.7 (11.5)	41.2 (13.2)
Gender	*Male*	1,087 (68.2)	465 (67.3)	622 (68.8)
	*Female*	508 (31.9)	226 (32.7)	282 (31.2)
WIMD 2019 quartile	*1 (Most dep.)*	801 (50.2)	353 (51.1)	448 (49.6)
	*2*	405 (25.4)	189 (27.4)	216 (23.9)
	*3*	256 (16.1)	101 (14.6)	155 (17.2)
	*4 (Least dep.)*	133 (8.3)	48 (7.0)	85 (9.4)
Local Health Board	*1*	413 (25.9)	184 (26.6)	229 (25.3)
	*2*	349 (21.9)	157 (22.7)	192 (21.2)
	*3*	306 (19.2)	133 (19.3)	173 (19.1)
	*4*	188 (11.8)	74 (10.7)	114 (12.6)
	*5*	124 (7.8)	53 (7.7)	71 (7.9)
	*6*	30 (1.9)	18 (2.6)	12 (1.3)
	*7*	185 (11.6)	72 (10.4)	113 (12.5)
Total		1,595 (100.0)	691 (43.3)	904 (56.7)

The median time-to-vaccination was 196 days (95% CI.: 184–209) for the study cohort, compared to 141 days (95% CI.: 141–141) among the matched adult population of similar individual and residential characteristics. The cumulative incidence of vaccination after 350-days of follow-up among the study cohort was 60.4% (95% CI.: 57.8–62.8), compared to 81.4% (95% CI.: 81.3–81.5) among the matched adult population.

**Fig. 1:**
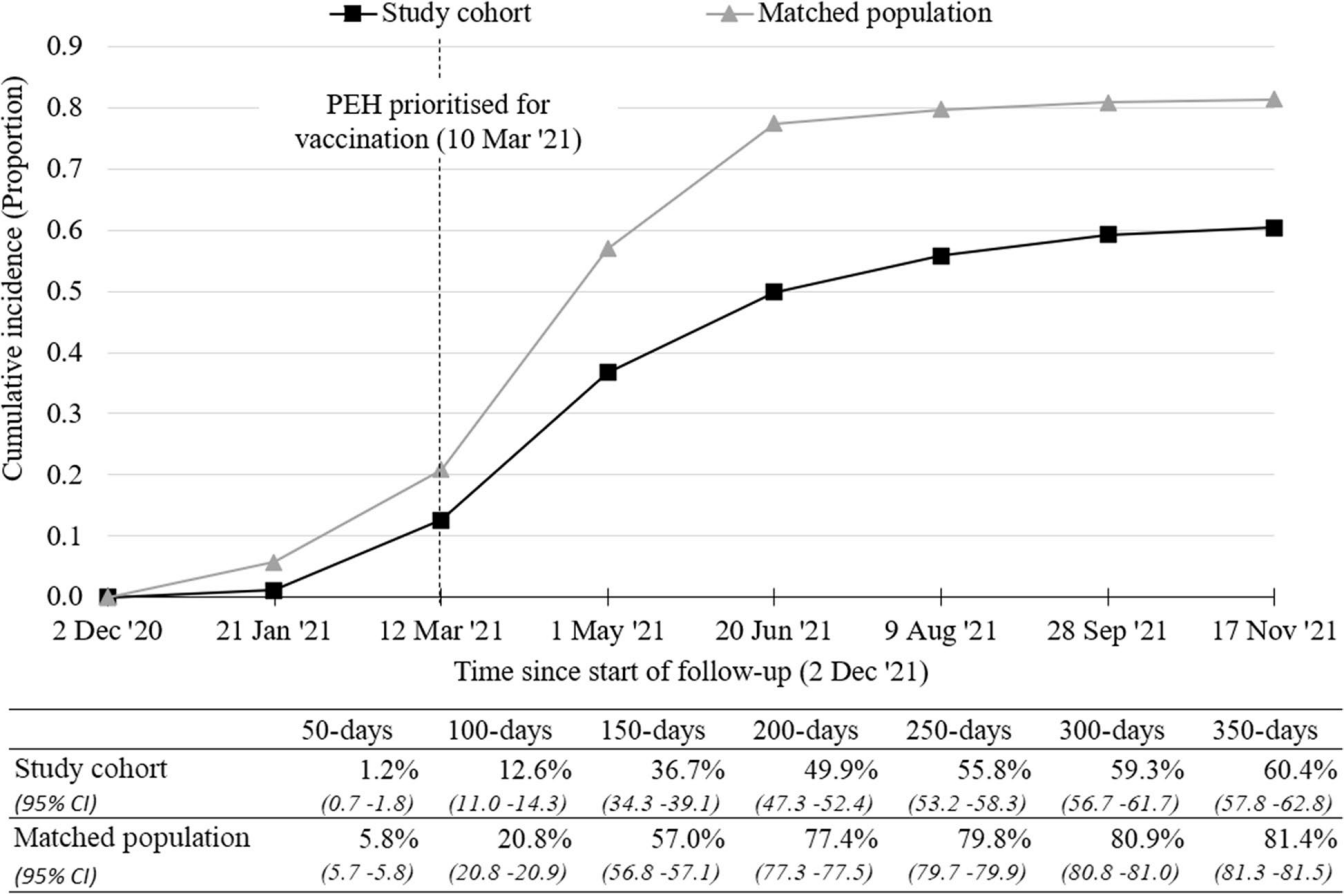
Cumulative incidence of receipt of first vaccine dose, measured at 50-day increments

Plotting cumulative incidence over time in Fig. 1, highlights that at all points from the start of follow-up, the study cohort had lower cumulative incidence of vaccination than the matched adult population in Wales. During the early stages of the vaccine programme, after 50-days follow-up (21 Jan 2021), the difference in cumulative incidence between the study cohort and matched adult population was roughly 5% points. After 100-days follow up (12 March 2021), vaccine inequality increased, reaching its greatest difference at 200-days (20 June 2021) of roughly 28% compared to adults of similar characteristics. From 250-days follow-up (9 August 2021), vaccine inequality declined slightly as cumulative incidence amongst the adult population in Wales plateaued, enabling the slow increase in cumulative incidence among the study cohort to reduce the vaccine inequality gap. However, by 300-days (28 September 2021), cumulative incidence appeared to be plateauing among the study cohort, at ~ 60%.

## Discussion

Almost a year into the vaccination programme in Wales, roughly 40% of people recently experiencing homelessness identified in this study had not received their first coronavirus vaccination, at a time when people in Wales were beginning to be prioritised for their booster vaccines. Compared to cumulative incidence among the adult population in Wales of similar characteristics, there was a ~ 20% point difference in cumulative incidence of vaccination. The scale and direction of this vaccination inequality is in line with international evidence based on population level analyses that include wider population comparators [23, 24]. Drawing parallels to the international literature, we now hypothesise potential individual and structural reasons for the lower rates of vaccination among the study cohort. Where relevant, we consider the possible impact the public health approach to vaccination prioritisation in Wales may have had in relation to the observed vaccine rates.

Despite international studies finding high levels of willingness to receive the coronavirus vaccine among people with experiences of homelessness (> 60%), they have expressed hesitancy, largely related to a distrust in the vaccine itself and officials/governments providing vaccines [38–42]. Though similar reasons for vaccine hesitancy are also found among people not experiencing homelessness [43], concerns may be more acute among people with experiences of homelessness due to previous negative interactions with ‘officials’ and services. For example, qualitative studies of interactions with healthcare services prior to the pandemic suggest people can be subject to discrimination because of their homelessness [44, 45]. The concentration of factors associated with hesitancy among people with experiences of homelessness may have contributed to lower vaccination rates seen in this group. However, one study from France found levels of hesitancy among people in homeless shelters to be comparable to that among the general population [46]. In addition to individual reasons for not being vaccinated, structural barriers to accessing local health and social care services have been raised by people with experiences of homelessness [47], which may have contributed to lower vaccine uptake.

A lack of permanent address can make registering with a primary care provider difficult, and, as vaccinations in Wales were offered using information largely derived when people registered with primary healthcare providers, people with experiences of homelessness may have been excluded from the initial offer. It is also conceivable that offer letters to attend vaccination centres were sent to addresses where people experiencing homelessness no longer resided. Welsh Government guidance that vaccines should be brought to people experiencing homelessness, i.e., in hostels, shelters, or food service locations, should have ameliorated some of the issues with the offer and physical access to the vaccine. However, early evidence on prioritisation activities from the housing-related support sector in Wales indicated that people with experiences of homelessness were primarily being directed to mass vaccination centres. Where vaccine outreach did occur, the approach was ad hoc rather than being consistent across Wales (Personal correspondence, Cymorth Cymru).

An important limiting factor to outreach in Wales may have been the lack of additional ringfenced funding to facilitate this work, with the cost of outreach for under-served groups being estimated at an additional £15 per/dose delivered [48]. This is a not-insignificant cost to be borne by healthcare providers, and third sector organisations which often play a key role in supporting people with experiences of homelessness to access health and social care in the UK [49]. This absence of additional funding is in stark contrast to the Welsh Government’s interventionist response to re-housing people experiencing homelessness at the start of the pandemic, which included detailed guidance, changes to legislation, and an unprecedented influx of funding for Local Authorities (~£10 million).

Given the pace at which the COVID-19 vaccination programme was rolled out across Wales, addressing vaccine inequality required the real-time identification and monitoring of population segments with low uptake [50]. However, the monitoring seen earlier in the pandemic in relation to rehousing people experiencing homelessness was absent in relation to their vaccine prioritisation. Indeed, this study represents the first time this issue has been enumerated at a population level in Wales. The lack of active monitoring of vaccine uptake among people with experiences of homelessness is in part due to a lack of a national individual level data collection in Wales related directly to homelessness. This study’s use of ‘non-housing’ data to flag people with recent experiences of homelessness was a pragmatic solution to facilitate population level analysis. However, adopting this methodology poses limitations.

Findings relate to people recently accessing health and substance use services at the more ‘extreme’ end of the homelessness spectrum. The study cohort does not therefore reflect homelessness in its broadest sense, particularly people accessing local authority support which include families known to have lower support needs [51]. However, the methodology for identifying people experiencing homelessness makes this study’s findings of vaccine inequality of greater interest. Having been recent contacts with public services, the study cohort should have been more easily identifiable for prioritisation by service providers. That the cumulative incidence of vaccination among the study cohort did not track that of the matched adult population after the date of prioritisation, indicates that the vaccine programme was not able to fully overcome structural and individual limiting factors.

As housing instability and homelessness are closely associated, breaks in residential histories identified using the Welsh Demographic Service could potentially have been miss-classified as migration out of Wales. We have attempted to account for this potential miss-classification through a 30-day grace period, i.e., if a gap in residential history was less than or equal to 30-days the person was considered not to have migrated out of Wales. Future research should examine the use of techniques for incomplete observation times, such as interval censored methods [52], to account for the increased housing instability of people with experiences of homelessness and other populations who are likely to be transitory.

A final limitation when interpreting these results comes from missed linkages to vaccine outcomes. A small percentage of vaccination records were not available for linkage due to a missing linkage field (0.2%). When monitoring vaccine uptake among larger segments of the population, i.e., older people [26, 34], missed linkages are residual and unlikely to affect outcomes. However, when engaging in vaccination equality research with vulnerable groups often at the margins of society, and at increased likelihood of not being identified in population registers, i.e., as they are not registered with a GP, missed linkages may be problematic. Missed linkages to vaccine records potentially led to a reduced ability to detect vaccination outcomes in this study. As missingness is likely to affect other studies of vaccine inequality among vulnerable groups, further exploration is required to understand the nature of missed matches.

## Conclusions

This study is important as it represents the first evidence of COVID-19 vaccination coverage among people recently experiencing homelessness in Wales. Based on the apparent inequality in COVID-19 vaccinations, this study has implications for future vaccination programmes for homeless populations and other socially vulnerable groups. We argue that a more interventionist approach with adequate real-time monitoring was required to boost low vaccine uptake among the homeless population, much like the response to re-housing people experiencing homelessness at the start of the pandemic. Future guidance would benefit from specifically addressing how clinicians and people working with homeless populations should identify and engage individuals for vaccination. Furthermore, additional ringfenced funding for outreach and engagement is vital, particularly when engaging people experiencing homelessness, where charities bear the brunt of service provision, in a sector that is historically under-funded, and where services are more-often-than not already stretched to capacity. The potential health implications of slower and less widespread vaccination of people experiencing homelessness in Wales should be the subject of future research.

## Data Availability

The linked data set generated as part of this analysis is not publicly available due to data sharing agreements limiting access to the research team only. However, the data sources used to create the linked dataset are listed in this publication and can be accessed via the Secure Anonymised Information Linkage (SAIL) Databank. To make enquiries into accessing data, please contact the SAIL Databank via the contact section of their webpage at https://saildatabank.com/contact/.

## Abbreviations

ALF: Anonymised Linkage Field
CI: Confidence interval, 95%
LHB: Local Health Board
NHS: National Health Service
RALF: Residential Anonymised Linkage Field
SAIL: Secure Anonymised Information Linkage Databank
SD: Standard deviation
WIMD: Welsh Index of Multiple Deprivation
